# Cinnamon Oil-Loaded Nanoliposomes with Potent Antibacterial and Antibiofilm Activities

**DOI:** 10.3390/molecules28114492

**Published:** 2023-06-01

**Authors:** Neveen M. Ellboudy, Bassma H. Elwakil, Marwa M. Shaaban, Zakia A. Olama

**Affiliations:** 1 Department of Botany & Microbiology, Faculty of Science, Alexandria University, Alexandria 21568, Egypt; 2 Department of Medical Laboratory Technology, Faculty of Applied Health Sciences Technology, Pharos University in Alexandria, Alexandria 21500, Egypt; 3 Department of Pharmaceutical Chemistry, Faculty of Pharmacy, Alexandria University, Alexandria 21568, Egypt

**Keywords:** cinnamon oil, antibacterial, antibiofilm, nanoliposome, colistin, combination study

## Abstract

Despite recent scientific advances, the global load of bacterial disease remains high and has been established against a backdrop of increasing antimicrobial resistance. Therefore, there is a pressing need for highly effective and natural antibacterial agents. In the present work, the antibiofilm effect provided by essential oils was evaluated. Of these, cinnamon oil extract showed potent antibacterial and antibiofilm activities against *Staphylococcus aureus* at an MBEC of 75.0 µg/mL. It was revealed that benzyl alcohol, 2-propenal-3-phenyl, hexadecenoic acid, and oleic acid were the major components of the tested cinnamon oil extract. In addition, the interaction between the cinnamon oil and colistin showed a synergistic effect against *S. aureus*. Cinnamon oil that had been combined with colistin was encapsulated by liposomes to enhance the essential oil’s chemical stability, demonstrating a particle size of 91.67 nm, a PDI of 0.143, a zeta potential of −0.129 mV, and an MBEC of 50.0 µg/mL against *Staphylococcus aureus*. Scanning electron microscopy was employed to observe the morphological changes in the *Staphylococcus aureus* biofilm that was treated with the encapsulated cinnamon oil extract/colistin. As a natural and safe option, cinnamon oil exhibited satisfactory antibacterial and antibiofilm performance. The application of liposomes further improved the stability of the antibacterial agents and extended the essential oil release profile.

## 1. Introduction

Biofilms are composed of microbial cells that have collected on surfaces and that are enclosed by a matrix formed primarily from polysaccharide materials. Biofilm-associated organisms differ from their planktonic (freely suspended) counterparts [1]. The bacteria in biofilms exhibit enhanced resistance to antibiotics. This results in persistent and stubborn infections, owing to their long-term viability. *Staphylococcus aureus* is prominent among the pathogenic microorganisms that can form biofilms [2].

The essential oils (Eos) derived from aromatic plants have several applications in complementary medicine and offer potent biological activities [3]. Essential oils are plant-based secondary metabolites that have a volatile nature, a soothing odor, and a lower density than water. Owing to their low toxicity, interest in the health benefits and pharmacological applications of essential oils has increased. Upon direct incorporation into edible products, essential oils demonstrate multiple beneficial activities, including antioxidant and antimicrobial behavior. As a consequence, the usage of essential oils has continued to rise in the fields of food and medicine. Essential oils are basically natural extracts composed of phytochemicals and mostly demonstrate biological properties; they present a rich source of active compounds. Roughly 3000 kinds of essential oils are known, of which 300 essential oils are currently being utilized in various industries. Many modern and traditional methods and techniques are used for the extraction of essential oils, such as hydrodistillation, steam distillation, ultrasound, ohmic- and microwave-assisted hydrodistillation, and supercritical fluid extraction [4]. Cinnamon has been employed as a spice for flavoring, as well as in medical applications for the treatment of diarrhea and other digestive system problems [5]. Moreover, cinnamon oil has been classified as a substance that is generally recognized as safe (GRAS) for food preservation by the US Food and Drug Administration (FDA) [6]. The main compounds that are present in cinnamon essential oil are cinnamaldehyde and eugenols, which are the reason for their strong antimicrobial activity. The phenolic substances present in cinnamon essential oil are the main source of its antioxidant, anticancer, antidiabetic, and anti-inflammatory activities. Cinnamaldehyde is an electronegative molecule that plays an important role in the biological processes that enable a reduction in food-borne bacteria [7]. Low microbial resistance towards EOs may be a result of their currently limited usage in clinical practice, due to their volatility, oxidation sensitivity, biological fluctuations, chemical instability, and low solubility. Therefore, a well-designed delivery system for essential oils is a necessity [8]. Many novel methods have been developed to enhance the stability and bioavailability of cinnamon oil. One of these methods involves the use of liposomal encapsulation to prevent the loss of the oil’s active ingredients during processing and storage. Liposomes, or spherical lipid bilayer vesicles, are formed by hydrophilic and lipophilic groups with an aqueous core and a hydrophobic outside layer. Naturally safe constituents (such as lecithin and cholesterol) are the main components of conventional liposomes. Liposomes have many advantages, including their bioavailability and biocompatibility, their ability to facilitate the solubilization of insoluble compounds, and their sustained-release profiles, which make them excellent EO carriers [9]. With the use of liposome encapsulation, the volatility and chemical instability of essential oils can be reduced without changing their chemical ingredients. Moreover, EO-loaded nanoliposomes have shown valuable anti-biofilm [10], anti-quorum sensing [11], and antibacterial [12] properties. Liposomes can also improve the antimicrobial activity of essential oils, mainly due to their subcellular size, which can strengthen the passive absorption mechanism of cells and reduce the transport resistance of materials [10]. Current research focuses on developing and evaluating novel drug delivery methods that are based on herbal medicine nanoliposomes. Hence, the present study has been conducted to identify the new antimicrobial and antibiofilm activities of cinnamon-oil-loaded nanoliposomes against multi-drug-resistant pathogens.

## 2. Results and Discussion

### 2.1. Antimicrobial and Antibiofilm Activities of Specific Essential Oil Extracts

A range of essential oils, namely cinnamon, ginger, fennel, lavender, rosemary, lemon, geranium, and tea tree oils, were tested for possible antimicrobial activity against various MDR bacteria, using the disc-diffusion method. The data shown in Table 1 reveal that cinnamon oil was the most potent essential oil extract, followed by tea tree oil, while the lowest level of activity was noted for the ginger oil extract. The highest inhibition zone diameters of 31.5 mm and 31 mm were recorded against *S. aureus* and *P. aeruginosa*, respectively, as seen upon using cinnamon oil extract. Therefore, antibiofilm activity was assessed against *S. aureus* and *P. aeruginosa*. The results revealed that the lowest MIC, MBC, and MBEC values for the cinnamon oil extract were 50, 100, and 75 µg/mL, respectively, when used against *S. aureus* (Table 2). Hence, the biological activity of cinnamon oil extract (COE) against *S. aureus* was chosen for further analysis.

### 2.2. GC–MS Analysis of Cinnamon Oil Extract (COE)

The COE was prepared and analyzed using GC–MS (Figure S1). Benzyl alcohol, 2-propenal-3-phenyl, hexadecenoic acid, and oleic acid were identified as the major components of the extract being tested, with area percentages of 23.5, 52.1, 8.1, and 7.7%, respectively (Table S1). Adinew [13] extracted Ethiopian cinnamon bark essential oil via a process of hydrodistillation and identified the presence of 2-propenal, 3-phenyl (87.013%), eugenol (9.317%), O-methoxy cinnamic aldehyde (0.236%), α-muurolene (0.133%), naphthalene,1,2,3,4-tetrahydro-1,6-dime (0.195%), tricyclo [3.3.1.0 (2,8)], and nona-3,6-dien-9-on (0.173%). The main compounds in COE are 2-propenal and 3-phenyl, which have been reported as the compound that is most responsible for cinnamon bark’s aroma and therapeutic effect. 

### 2.3. Combination Effect of COE with Some Commonly Used Antibiotics

The interaction of COE with some commonly used antibiotics was evaluated against *S. aureus*, using the disc-diffusion method. The data shown in Table 3 reveal that the combined action of COE with colistin (COE/C) showed a synergistic effect against *S. aureus*. Combination therapy has been widely applied in the medical industry to combat multi-drug-resistant microbes [14]. As the public becomes more health-conscious, novel antimicrobials are being sourced from plant-based compounds, such as essential oils, due to their reduced side effects and cost-effectiveness when developed commercially [15]. Only synergistic combinations (FICI ≤ 0.5) have been investigated further regarding their mechanism of action. Si et al. found that 5 of the 11 antibiotic–oregano essential oil combinations demonstrated synergism, whereas the other combinations interacted additively [16]. Van Vuuren et al. [17] examined 25 synergistic essential oil–antibiotic combinations out of 72; of these, 65% demonstrated additivity. Karpanen et al. [18] found that all four combinations of thymol, a plant secondary metabolite, and antibiotics only worked against MRSA. Chovanová et al. [19] performed another antimicrobial screening against MRSA and found that 50% of the plant extracts tested showed synergism with the antibiotic oxacillin, while the other 50% of the extracts being tested interacted additively. In addition, Yap et al. [20] tested 35 combinations of essential oils and antibiotics for synergism against the multi-drug-resistant pathogen *Escherichia coli*. Of these, only five combinations showed synergism, while the other 30 interacted additively [20]. Due to their high fractional concentrations, none of these synergistic combinations have been tested in clinical trials [21]. Two of the five combinations mentioned in [18] were studied for their modalities of action [22,23]. Yang et al. [24] combined cinnamon oil and meropenem, whereupon the FICI value reached 1 against *K. pneumoniae*, with a 64-fold FICI reduction in the meropenem dosage.

### 2.4. Synthesis and Characterization of Nanoliposomes

**Figure 1:**
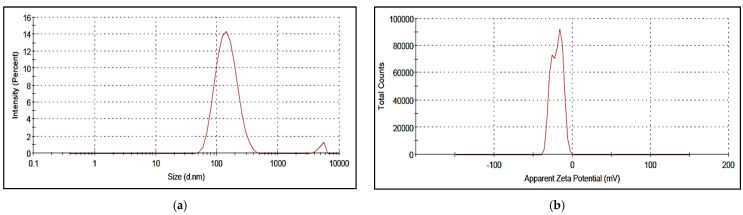
Zeta size (**a**) and zeta potential (**b**) of the synthesized nanoliposomes of COE/colistin 5.

**Figure 2:**
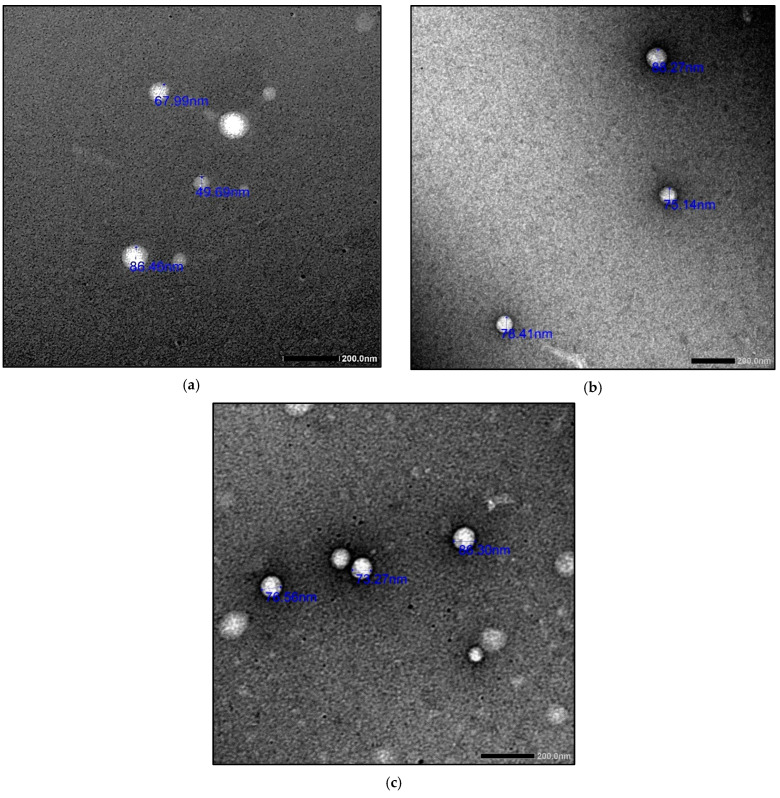
TEM images of the synthesized nanoliposomes: COE 5 (**a**), colistin 5 (**b**), and COE/colistin 5 (**c**).

The different COE/colistin-loaded liposomes were synthesized using half-MIC, MIC, and double-MIC values (COE (2, 5, and 10 µg) and colistin (2 and 5 µg), in addition to combined COE and colistin (2 µg COE/2 µg colistin and 5 µg COE/5 µg colistin)). Interestingly, the characterization of the synthesized liposomal formulations (Table 4) revealed that all the nanoformulations exhibited optimum particle sizes ranging from 88.44 to 156.6 nm, with acceptable PDI values in the range of 0.129–0.338. The COE-loaded liposomes 2 had the smallest vesicle size of 91.67 nm among all the loaded liposomes, with a PDI value and zeta potential of 0.143 and −0.129, respectively. Moreover, the colistin-loaded liposomes (2 and 5 µg) possessed comparably low particle sizes, with good PDI and zeta potential values. In the case of the samples combining COE and colistin, the COE/colistin-loaded liposomes (5 µg each) exhibited the smallest particle size, with a PDI value and zeta potential of 0.289 and −19.9, respectively (Figure 1 and Figure 2 and Figure S2 in the Supplementary Materials).

As suggested by Cui et al. [25], liposomes with smaller particle sizes (<200 nm) can achieve a high utilization ratio in terms of the entrapped compound. The surface zeta potential is a vital parameter that characterizes the dispersibility and stability of liposome systems. The higher zeta potential provides a repelling force between the particles, thus increasing the stability of the liposomes [26].

#### FT-IR Analysis

**Figure 3:**
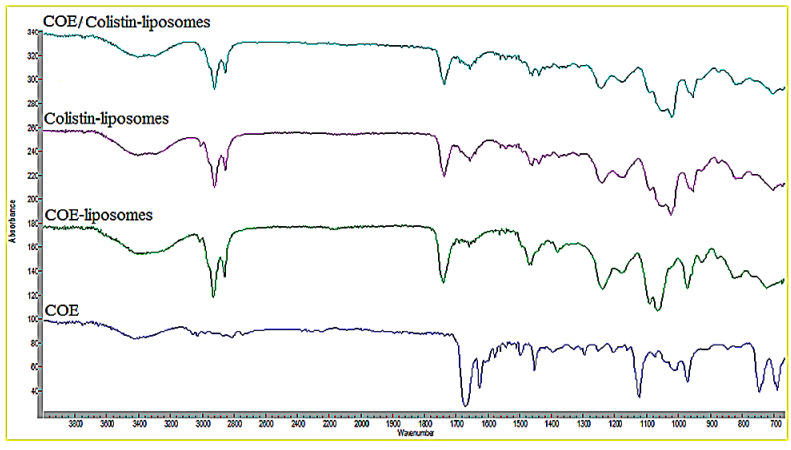
FT-IR analyses of the COE, COE-loaded liposomes (5 mg/mL), colistin-loaded liposomes (5 mg/mL), and COE/colistin-loaded liposomes (5 mg/mL each).

FT-IR spectroscopy was utilized to evaluate the structural and functional group information regarding correlations with the prepared nanoparticles. The FT-IR spectra of the COE, COE-loaded liposomes, colistin-loaded liposomes, and COE/colistin-loaded liposomes are illustrated in Figure 3. The formation of the loaded liposomes was confirmed by comparing the spectrum of the loaded liposomes with those of the individual components. The FT-IR spectrum of the COE showed a broad band at around 3430 cm^−^^1^ corresponding to the O–H group, in addition to characteristically intense bands at 1669 cm^−^^1^ and 1624 cm^−^^1^, corresponding to cinnamaldehyde C=O and the aromatic C=C groups, respectively. Moreover, the FT-IR analysis of the COE-loaded liposomes revealed two characteristically sharp bands at 2923 and 2852 cm^−^^1^, which correspond to the lipoid CH_2_ groups, in addition to a characteristically intense band at 1734 cm^−^^1^ corresponding to the lipoid C=O group. The FT-IR analysis also showed the disappearance of the C=O band (at 1669 cm^−^^1^) of the COE, which could be a sign of H-bonding, suggesting the presence of some weak physical interactions between the COE carbonyl group and the lipoid phosphate group [27]. Furthermore, the FT-IR analysis of the colistin-loaded liposomes showed a very broad band at around 3443 cm^−^^1^, corresponding to the lipoid O–H group overlapping with the NH band of colistin, in addition to the characteristically sharp bands at 1734 cm^−^^1^ and 1655 cm^−^^1^ corresponding to the C=O groups of the lipoid and colistin, respectively. However, some of the characteristic COE and colistin bands were masked in the nano-COE/colistin spectrum, suggesting the entrapment of the COE and colistin in the liposomal vesicles [28].

### 2.5. Antibacterial and Antibiofilm Activities of the Most Potent Nano-Formula Identified (COE/C)

**Figure 4:**
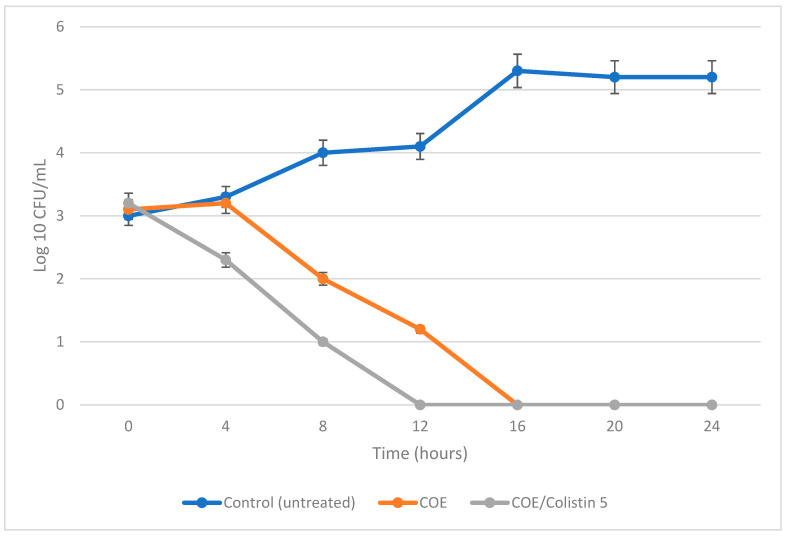
Bacterial lethality curve.

**Figure 5:**
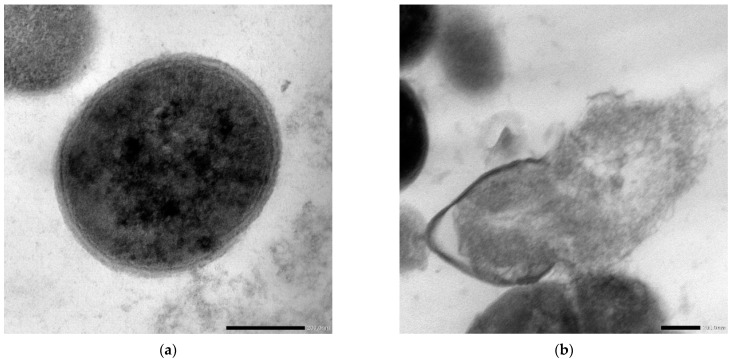
TEM images of *S. aureus*: control sample (**a**); sample treated with nano-COE/C (**b**).

The results of the present investigation revealed that the encapsulated nano-liposome/colistin “COE/colistin” (5 µg/mL each) showed potent antimicrobial and antibiofilm activities, with an IZ diameter, MIB, MBC, and MBEC values of 33.5 mm, 25, 50, and 50 µg/mL, respectively, when used against *S. aureus* (Table 5). The bacterial lethality curve presented in Figure 4 proves the superior effect of the prepared COE/colistin 5 with complete bacterial eradication after 12 h incubation. Further analyses that applied TEM were performed to assess the possible antimicrobial effect of the prepared nanoliposomes. The results presented in Figure 5 indicated that the nanoparticles were adsorbed to the cell surface, followed by cell penetration and interaction with the intracellular components, whereupon the cells turned into ghost cells. Zhang et al. [29] elucidated the mechanism of the antibacterial action of cinnamon oil when used against *E. coli* and *S. aureus* by observing any significant changes to cell permeability and membrane integrity. Zhang et al. [29] and Yap et al. [22] attributed the synergistic effect between cinnamon oil and meropenem to a postulated mode of action of cinnamon oil that eventually facilitated the influx of meropenem.

**Figure 6:**
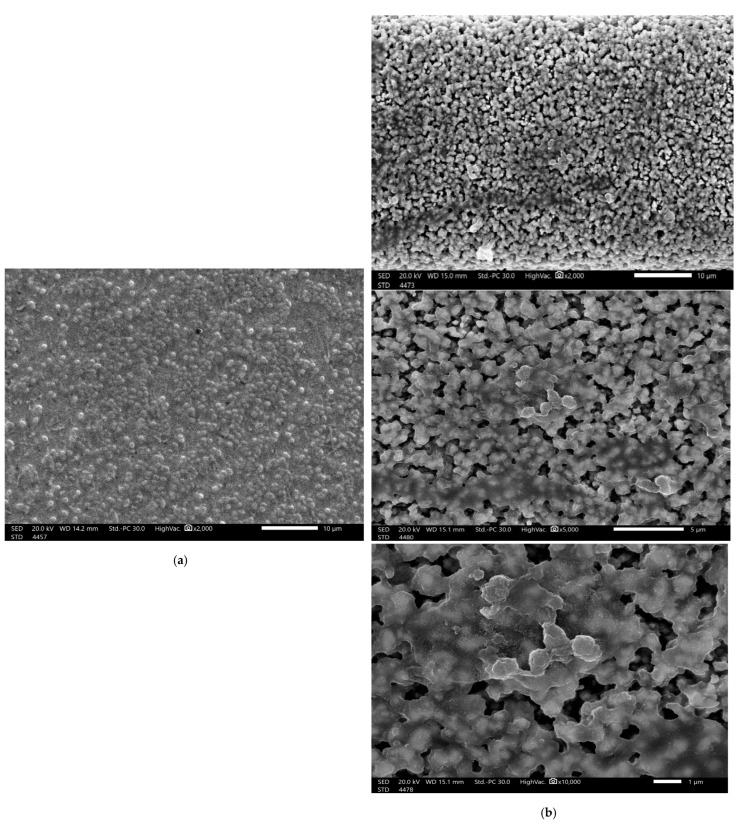
SEM images of *S. aureus*: control sample (**a**); biofilm treated with the potent formula, nano-COE/C (**b**).

The SEM analysis of the control biofilm revealed that the bacterial culture of *S. aureus* exhibited the expected normal cellular morphology, with smooth cell surfaces, and the bacterial cells were arranged in a noticeable exopolysaccharide matrix (Figure 6). When under the same growth conditions but in the presence of nanoliposome-encapsulated COE/C, the *S. aureus*-treated biofilm showed dramatically restricted bacterial colonization. The biofilm formation was markedly uneven, with visible changes in the cellular morphology, and microbial colonization was inhibited. It was clear that the prepared nanoformula had penetrated the pre-formed biofilm and eradicated the microbial cells. The degradation of established biofilms occurs either by inhibiting the growth of the formed bacteria or by detaching the living cells (Figure 6). Biofilm formation plays an essential role in enabling bacteria to invade the host’s immune defenses and in increasing antibiotic resistance, which encourages the persistence of microbial infections [30]. The highest antibiofilm and antimicrobial activities of essential oils are attributed to their high proportion of phenols and aldehydes. Hydrophobicity impacts EO activity by increasing cell permeability, resulting in cell leakage [31]. Most EOs, including cinnamon oil, cause lipopolysaccharides release, ATP balance change, quorum sensing inhibition, DNA disruption and internal cytoplasmic changes, such as the coagulation of cytoplasmic material [31].

## 3. Materials and Methods

### 3.1. Extraction of Bioactive Material

A conventional hydrodistillation method was used for the extraction of essential oil from *Cinnamomum verum* (cinnamon) bark, *Zingiber officinale* (ginger) roots, *Foeniculum vulgare* (fennel) leaves, *Lavandula angustifolia* (lavender) leaves, *Salvia rosmarinus* (rosemary) leaves, *Citrus limon* (lemon) peel, *Geranium sanguineum* (geranium) flowers, and *Melaleuca alternifolia* (tea tree) leaves [32].

### 3.2. Antimicrobial Effect of Essential Oil Extracts

Antimicrobial activity was carried out using the disc-diffusion method, according to CLSI guidelines [33]. By measuring the lowest inhibitory concentration (MIC) and the minimum bactericidal concentration (MBC), further antibacterial activity was evaluated [34].

### 3.3. Minimal Biofilm Eradication Concentration (MBEC)

The microbial cultures were grown overnight in Mueller Hinton and Saboraud Dextrose broth for bacterial and fungal strains, respectively. An overnight culture (of each strain under test) at 10^8^ CFU/mL was diluted to 1:100 with a fresh medium for the biofilm assays. The biofilm was allowed to grow for 48 h in a 96-well microtiter plate in both the absence and presence of the prepared oil extracts, which were tested one at a time. The wells were subsequently washed thoroughly with water to remove the free-floating and loosely adherent microbial cells, then the titer-plate wells were fixed with 2% sodium acetate and treated with 0.1 mL of crystal violet (0.4%) for 15 min [35].

### 3.4. GC–MS (Gas Chromatography–Mass Spectroscopy) Analysis

The chemical analysis and component identification of the oil extract using GC–MS analysis were assessed according to the method developed by Hamza et al. [36].

### 3.5. Combination Effect of the Most Promising Essential Oil with Commonly Used Antibiotics Using the Disc-Diffusion Method

Different antibiotics were selected for use in the present experiment, namely, vancomycin (VA, 30 μg), ampicillin/cloxacillin (AX, 10 µg), colistin (CT, 10 µg), cefuroxime (CXM, 30 µg), doxycycline (DO, 5 µg), ampicillin (AMP, 10 µg), erythromycin (E, 10 µg), cefoxitin (FOX, 30 µg), ceftazidime (CAZ, 30 μg), and cotrimoxazole (COT, 25 µg). The antibiotic discs were loaded with 20 µL of each extract (20 µg), one at a time, and were then placed on the surface of the inoculated Müeller–Hinton agar. When the combined effect was equal to the sum of the individual effects, the action was considered to be additive. Antagonism was considered to be present when the effect of the combined compounds was less marked than when the substances were individually applied. Synergism was considered to be present when the effect of the combined compounds was greater than the sum of the individual effects, while the absence of interaction was defined as indifference [37].

#### Checkerboard Assay

A checkerboard assay was performed via a two-fold dilution essay of each tested antibiotic and potent essential oil to determine the combinatory effects of the essential oils and colistin against *S. aureus*. In 96-well plates, 25 μL of the tested antibiotic and 25 μL of the potent essential oil were inoculated with 40 μL of the bacterial suspension (1 × 10^5^ CFU/mL) and were then incubated at 37 °C for 20 h. The combinatory relationship between the tested antibiotic and the potent essential oil was expressed in terms of the fractional inhibitory concentration index (FICI), as described previously by Lorian [38] and Yang et al. [24]: FICI ≤ 0.5 (synergistic); FICI > 0.5–4.0 (additive); FICI > 4.0 (antagonistic).

### 3.6. Synthesis of Liposome Nanoparticles

Lipoid S100 (75 mg) and cholesterol (18.75 mg), in addition to the requisite amounts of cinnamon oil extract (COE) and/or colistin (Table 6), were dissolved in absolute ethanol (1.5 mL). The resulting organic phase was injected by means of a syringe pump into 20 mL of distilled water undergoing magnetic stirring. Spontaneous liposome formation occurred as soon as the ethanolic solution came into contact with the aqueous phase. The liposome suspension was then maintained, with stirring, for 1 h at room temperature. Finally, the ethanol and part of the water were removed via rotary evaporation under reduced pressure [39].

### 3.7. Characterization of the Synthesized Nanoliposomes

The prepared nanoparticles were characterized using dynamic light scattering (DLS) techniques to determine the vesicle size, polydispersity index (PDI), and zeta potential and to conduct FT-IR analysis. The ultra-structure of the prepared nanoparticles was analyzed via transmission electron microscopy (TEM) [40].

### 3.8. Antimicrobial and Antibiofilm Activities of the Synthesized Nanoliposomes

The antimicrobial activity of the prepared nanoparticles was evaluated using the disc-diffusion method, minimal inhibitory concentration (MIC) and minimum bactericidal concentration (MBC) values, microbial lethality curve and a TEM examination of the microbially treated cells [40]. Moreover, antibiofilm activity was assessed using MBEC, as previously described by Dorgham et al. [41]. The fixed cells were oriented, mounted on aluminum stubs, and coated with gold before imaging took place [35]. The topographic features of the biofilms were visualized using SEM (JEOL JSM-6390LV, JOEL, Shanghai, China).

### 3.9. Statistical Analysis

The obtained data are presented as the mean value ± standard deviation (SD). The statistical significance was set at *p* < 0.05.

## 4. Conclusions

Increased antimicrobial resistance is considered a global crisis, resulting from antibiotic misuse and abuse over the years. Moreover, biofilm formation increases the microbial resistance by 1000 times and high percentages of nosocomial infections were due to biofilm forming pathogens. Hence, we aimed to combat some multi-drug resistant/biofilm forming microbes. In this study, the optimal synthesized nanoformula for liposome-encapsulated cinnamon oil and colistin (5.0 mg mL^−1^ each) was established. As a natural and safe spice, cinnamon oil exhibits an excellent level of antibacterial effect against *S. aureus* (individual cells) and *S. aureus* biofilm. The introduction of liposomes can significantly improve the stability of cinnamon oil in the elimination of *S. aureus* biofilms. The formulated nanoliposomes caused increased cell permeability, resulting in cell leakage. The concept explored in the present study may be useful in the future for delivering a variety of antimicrobials and antibiotics for use in the treatment of various microbial biofilm infections.

## Figures and Tables

**Table 1:** Antimicrobial activity of specific essential oils against MDR pathogens.

Tested Pathogens	Inhibition Zone Diameter (mm) ± SD							
	Cinnamon	Ginger	Fennel	Lavender	Rosemary	Lemon	Geranium	Tea Tree
*E. coli*	30.5 ± 6	6.0 ± 2	6.0 ± 0	6.0 ± 0	10.0 ± 2	6.0 ± 0	6.0 ± 0	6.0 ± 0
*S. aureus*	31.5 ± 3	6.0 ± 0	8.0 ± 1	6.0 ± 0	15.0 ± 2	23.0 ± 4	17.0 ± 2	21.0 ± 4
*E. faecalis* 1 *	19.0 ± 5	6.0 ± 0	15.0 ± 4	6.0 ± 0	8.0 ± 4	20.0 ± 2	12.0 ± 3	9.0 ± 2
*E. faecalis* 2 *	30.0 ± 6	6.0 ± 0	12.0 ± 2	6.0 ± 0	13.0 ± 2	6.0 ± 0	19.0 ± 1	29.0 ± 1
*K. pneumonia* 1 *	19.0 ± 2	6.0 ± 0	7.0 ± 1	15.0 ± 3	15.0 ± 2	6.0 ± 0	14.0 ± 1	24.0 ± 3
*K. pneumonia* 2 *	22.0 ± 1	6.0 ± 0	10.0 ± 1	6.0 ± 0	6.0 ± 0	6.0 ± 0	21.0 ± 2	22.0 ± 1
*P. vulgaris*	30.0 ± 2	6.0 ± 0	6.0 ± 0	6.0 ± 0	14.0 ± 4	6.0 ± 0	8.0 ± 2	28.0 ± 4
*P. aeruginosa*	31.0 ± 6	6.0 ± 0	6.0 ± 0	6.0 ± 0	6.0 ± 0	6.0 ± 0	8.0 ± 1	8.0 ± 3
*A. bauminii*	30.0 ± 2	6.0 ± 0	6.0 ± 0	6.0 ± 0	16.0 ± 3	6.0 ± 0	6.0 ± 0	12.0 ± 1
MRSA	21.0 ± 3	6.0 ± 0	8.0 ± 3	6.0 ± 0	12.0 ± 2	10.0 ± 2	12.0 ± 3	13.5 ± 1

MRSA: methicillin resistant *Staphylococcus areus*, * different numbers indicate different strain.

**Table 2:** The MIC, MBC, and MBEC of the various essential oils against the tested pathogens.

Oil Extracts	Tests	(µg/mL)	
		*S. aureus*	*P. aeruginosa*
Cinnamon	MIC	50.0	125.0
	MBC	100.0	250.0
	MBEC	75.0	250.0
Ginger	MIC	125.0	250.0
	MBC	250.0	350.0
	MBEC	375.0	400.0
Fennel	MIC	250.0	500.0
	MBC	400.0	1000.0
	MBEC	500.0	1000.0
Lavender	MIC	450.0	500.0
	MBC	500.0	1000.0
	MBEC	750.0	1000.0
Rosemary	MIC	500.0	1000.0
	MBC	750.0	1500.0
	MBEC	1000.0	1500.0
Lemon	MIC	125.0	500.0
	MBC	250.0	750.0
	MBEC	500.0	1000.0
Geranium	MIC	150.0	250.0
	MBC	300.0	500.0
	MBEC	500.0	1000.0
Tea tree	MIC	125.0	300.0
	MBC	250.0	500.0
	MBEC	500.0	750.0

**Table 3:** The combination effect of cinnamon oil extract with commonly used antibiotics.

	Antibiotics	Inhibition Zone Diameter (mm)	MIC (µg/mL)	FIC	FICI
Vancomycin	Alone	21.0 ± 4	32.0	2.0	6.0
	With COE	36.5 ± 6	64.0		
Ampicillin/Cloxacillin	Alone	8.0 ± 3	500.0	1.0	2.0
	With COE	38.0 ± 6	500.0		
Colistin	Alone	6.0 ± 0	16.0	0.125	0.4
	With COE	38.0 ± 3	2.0		
Cefuroxime	Alone	6.0 ± 0	32.0	2.0	5.0
	With COE	32.5 ± 2	64.0		
Doxycycline	Alone	30.0 ± 7	500.0	1.0	7.0
	With COE	31.0 ± 3	500.0		
Ampicillin	Alone	33.0 ± 2	500.0	1.0	6.0
	With COE	34.0 ± 1	500.0		
Ceftazidime	Alone	6.0 ± 1	32.0	0.5	1.0
	With COE	33.5 ± 3	16.0		
Erythromycin	Alone	30.0 ± 2	125.0	1.0	6.0
	With COE	34.5 ± 2	125.0		
Cotrimoxazole	Alone	15.0 ± 4	32.0	2.0	3.0
	With COE	35.5 ± 4	64.0		
Cefoxitin	Alone	6.0 ± 0	16.0	0.5	0.9
	With COE	29.5 ± 3	8.0		

**Table 4:** Characterization of the different nano-liposomes.

Formula	Particle Size (nm)	Polydispersity Index	Zeta Potential (mV)	Encapsulation Efficiency (%)
Blank liposomes	88.44	0.185	−12.80	NA
COE 2	91.67	0.143	−0.129	90.2
COE 5	129.7	0.209	−0.309	81.7
COE 10	124.8	0.129	−0.168	80.4
Colistin 2	137.2	0.221	−15.9	63.5
Colistin 5	132.8	0.338	−21.4	67.9
COE/Colistin 2	156.6	0.260	−22.8	83.8
COE/Colistin 5	150.0	0.289	−19.9	93.7

NA: not available.

**Table 5:** The antimicrobial and antibiofilm activities of nanoliposome-encapsulated COE/C.

Tested Nanoformula	IZ (mm)	MIC (µg/mL)	MBC (µg/mL)	MBEC (µg/mL)
COE/Colistin 5	33.5 ± 3	25.0	50.0	50.0

**Table 6:** The nanoliposome preparations and formulations.

Nanoformula	Lipoid S100 (mg/mL)	Cholesterol (mg/mL)	Cinnamon Oil Extract (COE) (µg/mL)	Colistin (µg/mL)
Blank liposomes	75.0	18.75	-	-
COE 2	75.0	18.75	2.0	-
COE 5	75.0	18.75	5.0	-
COE 10	75.0	18.75	10.0	-
Colistin 2	75.0	18.75	-	2.0
Colistin 5	75.0	18.75	-	5.0
COE/Colistin 2	75.0	18.75	2.0	2.0
COE/Colistin 5	75.0	18.75	5.0	5.0

## Data Availability

Data will be made available on request.

## Supplementary Materials

The following supporting information can be downloaded at: https://www.mdpi.com/article/10.3390/molecules28114492/s1, Figure S1: GC–MS chromatogram of cinnamon oil; Figure S2: Zeta size and potential of the synthesized nanoliposomes: COE 2 (2 µg/mL; a,b), COE 5 (5 µg/mL; c,d), COE 10 (10 µg/mL; e,f), Colistin 2 (2 µg/mL; g,h), Colistin 5 (5 µg/mL; i,j), COE/Colistin 2 (2 µg/mL each; k,l), and COE/Colistin 5 (5 µg/mL each; m,n); Table S1: The main predicted compounds of cinnamon oil extract structures.
