# Supplementary material for: Cinnamon Oil-Loaded Nanoliposomes with Potent Antibacterial and Antibiofilm Activities
Source: Molecules. 2023 Jun 1;28(11):4492. doi: 10.3390/molecules28114492 (PMC10254904; doi:10.3390/molecules28114492)
Supplement: Supplementary file 1 [file molecules-28-04492-s001.zip › molecules-2410345-supplementary.pdf]

Cinnamon-oil-loaded nanoliposomes with potent antibacterial and antibiofilm activities

Neveen M. Ellboudy[1], Bassma H Elwakil[2*], Marwa M. Shaaban[3], Zakia A Olama[1]

[1] Department of Botany & Microbiology, Faculty of Science, Alexandria University, Alexandria 21568, Egypt
[2] Department of Medical Laboratory Technology, Faculty of Applied Health Sciences Technology, Pharos University in Alexandria, Alexandria 21500, Egypt
[3] Department of Pharmaceutical Chemistry, Faculty of Pharmacy, Alexandria University, Alexandria 21568, Egypt
* Correspondence: bassma.hassan@pua.edu.eg

Supplementary data

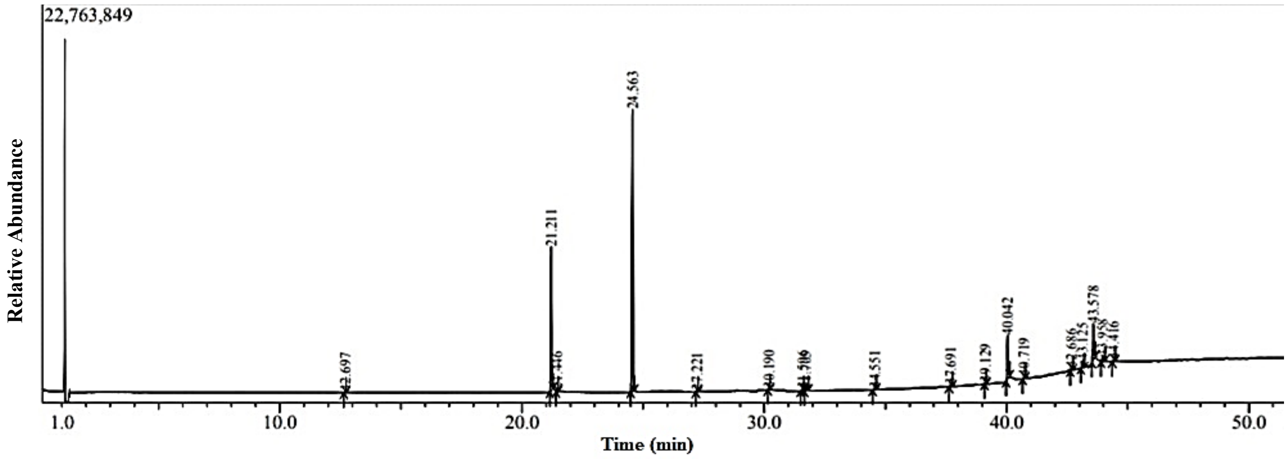

Figure S1. GC-MS chromatogram of cinnamon oil.

Table S1. The main predicted compounds of cinnamon oil extract structures.

Peak	Retention time	Area (%)	Name
1	12.6	0.3	Benzaldehyde
2	21.2	23.5	Benzyl alcohol
3	21.4	0.3	2-propenal, 3-phenyl
4	24.5	52.1	2-propenal, 3-phenyl
5	27.2	0.3	3-allyl-6-methoxyphenol
6	30.1	0.2	Diethyl phthalate
7	31.5	0.7	1,7-di(3-ethylphenyl)
8	31.7	0.5	1,7-di(3-ethylphenyl)
9	34.5	0.4	1,2-diphenyltetramethyldisilane
10	37.6	0.4	Bis-di(trimethylsiloxy)phenylsiloxy propane
11	39.1	0.4	Hexadecenoic acid, 1 hydroxymethane
12	40.0	8.1	n-Hexadecenoic acid
13	40.7	0.5	Bis-di(trimethylsiloxy)phenylsiloxy propane
14	42.6	0.7	9-Octadecanoic acid,1,2,3-propane
15	43.1	2.1	Octadecanoic acid
16	43.5	7.7	Oleic acid
17	43.9	1.2	Triphenylphosphine oxide
18	44.4	0.6	9,12-octadecadienoic acid (Z,Z)

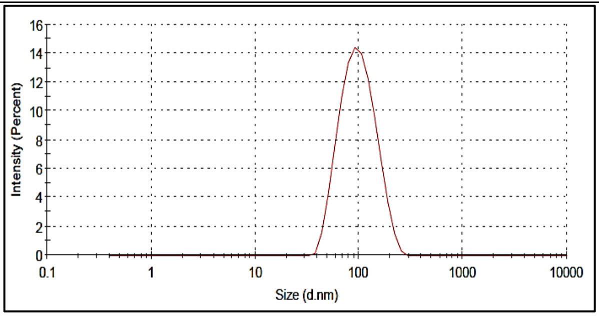
Intensity (Percent)
0 2 4 6 8 10 12 14 16
0.1 1 10 100 1000 10000
Size (d.nm)

(a)

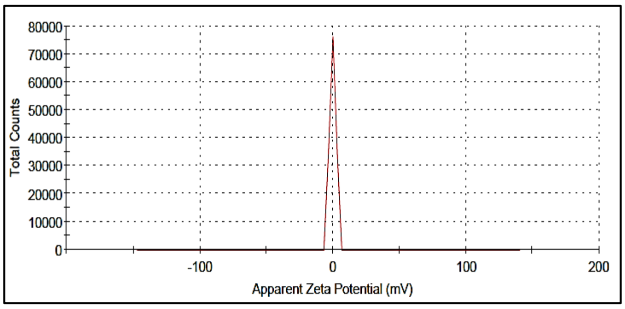
Total Counts
0 10000 20000 30000 40000 50000 60000 70000 80000
-100 0 100 200
Apparent Zeta Potential (mV)

(b)

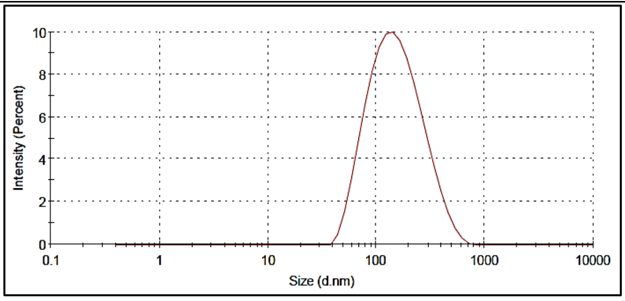
Intensity (Percent)
0 2 4 6 8 10
0.1 1 10 100 1000 10000
Size (d.nm)

(c)

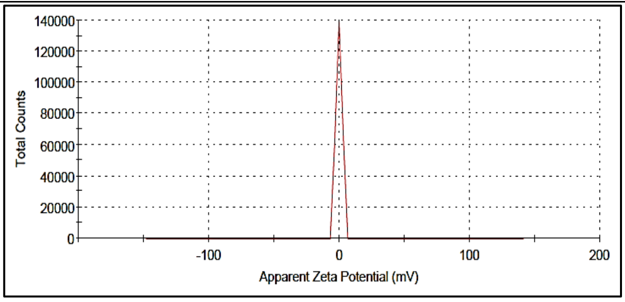
Total Counts
0 20000 40000 60000 80000 100000 120000 140000
-100 0 100 200
Apparent Zeta Potential (mV)

(d)

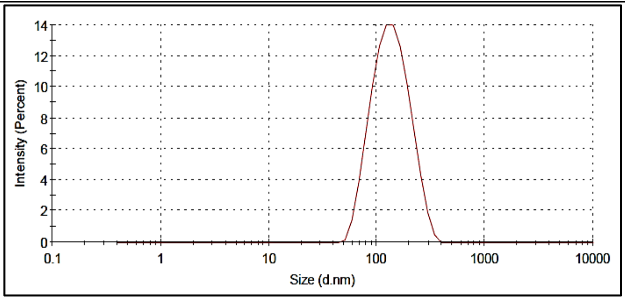
Intensity (Percent)
0 2 4 6 8 10 12 14
0.1 1 10 100 1000 10000
Size (d.nm)

(e)

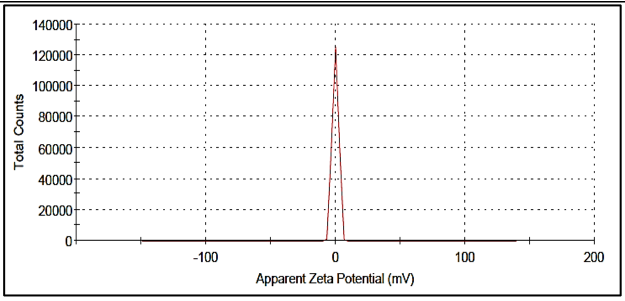
Total Counts
0 20000 40000 60000 80000 100000 120000 140000
-100 0 100 200
Apparent Zeta Potential (mV)

(f)

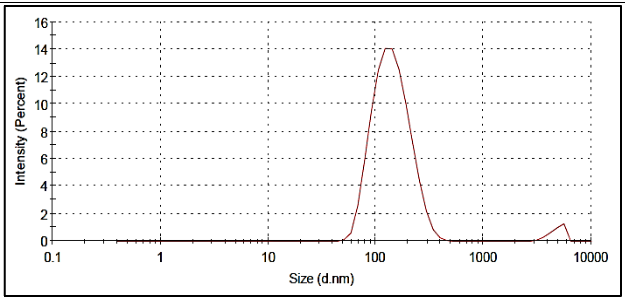
Intensity (Percent)
0 2 4 6 8 10 12 14 16
0.1 1 10 100 1000 10000
Size (d.nm)

(g)

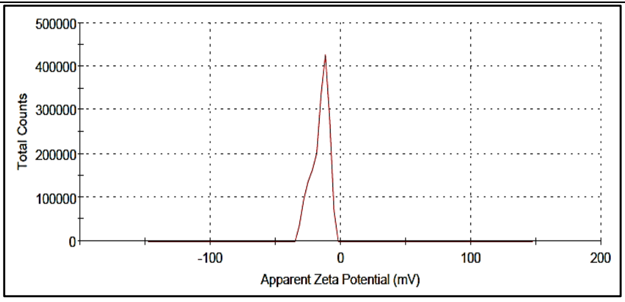
Total Counts
0 100000 200000 300000 400000 500000
-100 0 100 200
Apparent Zeta Potential (mV)

(h)

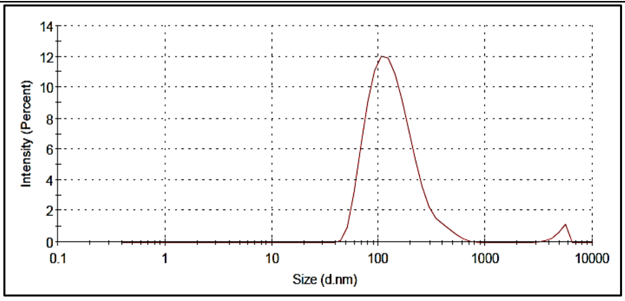
Intensity (Percent)
0 2 4 6 8 10 12 14
0.1 1 10 100 1000 10000
Size (d.nm)

(i)

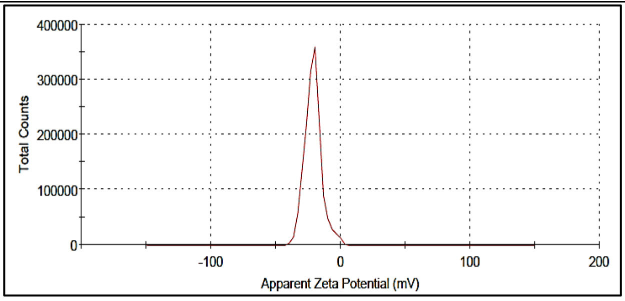
Total Counts
0 100000 200000 300000 400000
-100 0 100 200
Apparent Zeta Potential (mV)

(j)

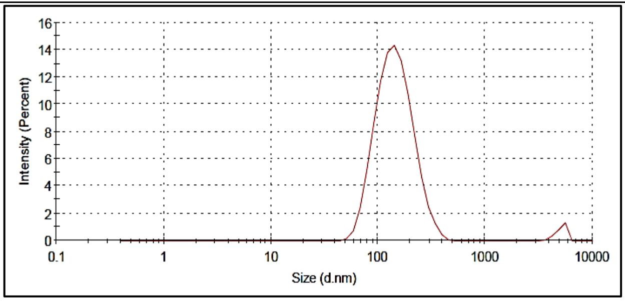

(k)

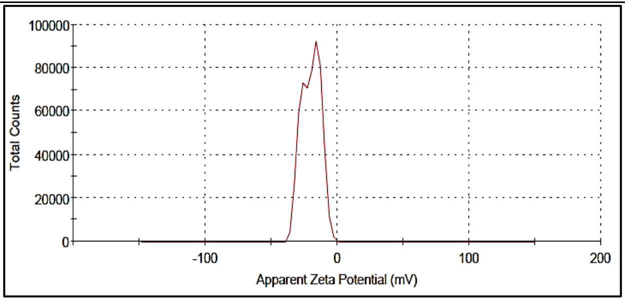

(l)

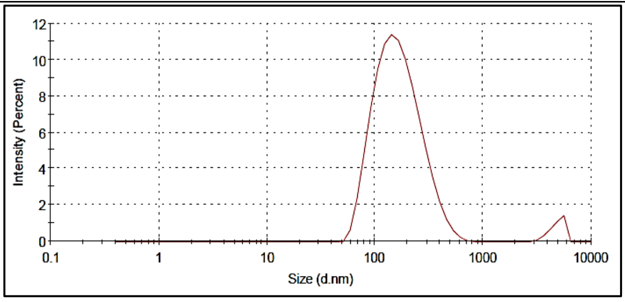

(m)

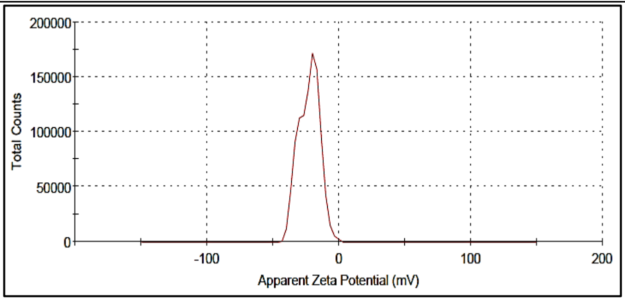

(n)

Figure S2. Zeta size and potential of the synthesized nanoliposomes: COE 2 (2 µg/mL; a, b), COE 5 (5 µg/mL; c, d), COE 10 (10 µg/mL; e, f), Colistin 2 (2 µg/mL; g, h), Colistin 5 (5 µg/mL; i, j), COE/Colistin 2 (2 µg/mL each; k, l) and COE/Colistin 5 (5 µg/mL each; m, n).
